# Autoantibodies to N-terminally Truncated GAD_65_(96-585): HLA Associations and Predictive Value for Type 1 Diabetes

**DOI:** 10.1210/clinem/dgab816

**Published:** 2021-11-08

**Authors:** Petra M Pöllänen, Taina Härkönen, Jorma Ilonen, Jorma Toppari, Riitta Veijola, Heli Siljander, Mikael Knip

**Affiliations:** 1 Pediatric Research Center, Children’s Hospital, University of Helsinki and Helsinki University Hospital, Helsinki, Finland; 2 Research Program for Clinical and Molecular Metabolism, Faculty of Medicine, University of Helsinki, Helsinki, Finland; 3 Immunogenetic Laboratory, Institute of Biomedicine, University of Turku, Turku, Finland; 4 Department of Pediatrics, Turku University Hospital, and Institute of Biomedicine and Centre for Population Health Research, University of Turku, Turku, Finland; 5 Department of Pediatrics, PEDEGO Research Group, Medical Research Center, Oulu University Hospital and University of Oulu, Oulu, Finland; 6 Tampere Center for Child Health Research, Tampere University Hospital, Tampere, Finland

**Keywords:** type 1 diabetes, prediction, HLA, GAD autoantibodies

## Abstract

**Objective:**

To evaluate the role of autoantibodies to N-terminally truncated glutamic acid decarboxylase GAD_65_(96-585) (t-GADA) as a marker for type 1 diabetes (T1D) and to assess the potential human leukocyte antigen (HLA) associations with such autoantibodies.

**Design:**

In this cross-sectional study combining data from the Finnish Pediatric Diabetes Register, the Type 1 Diabetes Prediction and Prevention study, the DIABIMMUNE study, and the Early Dietary Intervention and Later Signs of Beta-Cell Autoimmunity study, venous blood samples from 760 individuals (53.7% males) were analyzed for t-GADA, autoantibodies to full-length GAD_65_ (f-GADA), and islet cell antibodies. Epitope-specific GAD autoantibodies were analyzed from 189 study participants.

**Results:**

T1D had been diagnosed in 174 (23%) participants. Altogether 631 (83%) individuals tested positive for f-GADA and 451 (59%) for t-GADA at a median age of 9.0 (range 0.2-61.5) years. t-GADA demonstrated higher specificity (46%) and positive predictive value (30%) for T1D than positivity for f-GADA alone (15% and 21%, respectively). Among participants positive for f-GADA, those who tested positive for t-GADA carried more frequently HLA genotypes conferring increased risk for T1D than those who tested negative for t-GADA (77% vs 53%; *P* < 0.001).

**Conclusions:**

Autoantibodies to N-terminally truncated GAD improve the screening for T1D compared to f-GADA and may facilitate the selection of participants for clinical trials. HLA class II-mediated antigen presentation of GAD(96–585)-derived or structurally similar peptides might comprise an important pathomechanism in T1D.

The 65 kD isoform of glutamic acid decarboxylase (GAD) is a major type 1 diabetes (T1D)-associated autoantigen and as the primary autoantigen represents one of the 2 major projected disease endotypes leading to T1D (1). Autoantibodies to GAD (GADA) are commonly used to identify patients with autoimmune diabetes and individuals at risk for T1D. However, they are also detected in many nondiabetic subjects with low risk to develop the disease. This has generated the need to discover more specific GADA assays for estimation of the risk for T1D.

Autoantibodies to N-terminally truncated GAD (t-GADA) comprising amino acids 96-585 have been reported to identify more specifically at-risk relatives of patients with T1D than autoantibodies to full-length GAD (f-GADA) (2). In contrast, isolated positivity for autoantibodies to the N-terminal epitope of GAD confers no increased risk for T1D (3,4). Accordingly, identifying the disease risk associated with GAD epitope-specific immune responses might provide improved tools for the prediction of T1D and for the selection of participants into prospective studies, including diabetes prevention trials.

At present, the identification of individuals at high risk for clinical T1D is largely based on the screening for genetic risk factors and islet autoantibodies (5,6). The genetic risk for T1D is mainly defined by the combination of 2 inherited human leukocyte antigen (HLA) class II haplotypes within an individual and can be classified in an order ranging from strong protection to highly increased risk (7). The highest HLA risk for T1D is mediated by the combination of the high-risk *HLA-DR3-DQ2* and *DR4-DQ8* haplotypes (7). However, how the HLA genotypes conferring increased risk for T1D relate to GAD epitope-specific autoantibodies has not been studied previously. An association has been previously reported between the *HLA-DQ8* haplotype and t-GADA (8).

In this study we combined autoantibody and genetic data from 4 studies carried out in populations with a variable distribution of HLA genotypes to evaluate whether t-GADA identified more specifically individuals at risk for T1D than f-GADA and whether positivity for t-GADA or GAD epitope-specific autoantibodies were associated with HLA genotypes predisposing to T1D. We also explored the association between t-GADA on one hand and positivity for multiple autoantibodies and islet cell antibodies (ICA) on the other hand. Moreover, we assessed the relationship between f-GADA titer and t-GADA positivity.

## Materials and Methods

### Study Participants

The current study comprises data from 760 individuals (53.7% males) from 6 cohorts including newly diagnosed children with T1D, children with HLA-conferred susceptibility to T1D, first-degree relatives (FDRs) of children with T1D, and GADA-positive children from the general population.

#### Finnish Pediatric Diabetes Register

The Finnish Pediatric Diabetes Register (FPDR) and Sample Repository is a nationwide cross-sectional project comprising information on more than 90% of children under the age of 16 years in Finland diagnosed with diabetes since 2002 and from their biological FDRs (ie, parents and siblings) (9). The register covers data on clinical and metabolic variables at the diagnosis of T1D, HLA-conferred susceptibility to T1D, diabetes-associated autoantibodies, and questionnaire-based family history of autoimmune diseases. All autoantibody samples from the FPDR have been obtained close to the time of the diagnosis in the index child. No prospective follow-up of the participants was carried out.

The current study includes 90 FPDR children diagnosed with T1D and 365 other FDRs. The FPDR participants were included in the current study population based on varying concentrations of f-GADA and/or ICA at the diagnosis of the index children. Among the FDRs, 2 siblings aged 12 and 18 years had later progressed to T1D. Data on progression to T1D were not available for the other family members.

#### Type 1 Diabetes Prediction and Prevention **study**

The Type 1 Diabetes Prediction and Prevention (DIPP) study is a prospective birth cohort study running in the University Hospitals in Turku, Oulu, and Tampere, Finland. Cord blood samples from newborn infants born in the participating hospitals are screened for the major T1D-associated HLA-DR-DQ haplotypes based on written informed parental consent (10). The study design has been described in detail in earlier DIPP reports (5,11). In summary, HLA-predisposed children are monitored for signs of islet autoimmunity every 3 to 6 months up to the age of 2 years and thereafter every 6 to 12 months, except in the case of participants who have seroconverted to positivity for at least 1 islet autoantibody. They are seen every 3 months after the seroconversion. ICA, f-GADA, and autoantibodies to insulin (IAA), islet antigen-2 (IA-2A), and zinc transporter 8 (ZnT8A) are analyzed as markers of islet autoimmunity.

In children born before the end of 2002, the primary screening in the DIPP study was ICA-based. If a participant developed ICA positivity or clinical T1D, all their earlier and subsequent samples were also analyzed for IAA, f-GADA, and IA-2A. Since 2003, all samples from all new participants have been analyzed for ICA, IAA, f-GADA, and IA-2A. Also, all available samples from the first 1006 children recruited in 1994-1997 have been analyzed directly for ICA, IAA, f-GADA, and IA-2A and later for ZnT8A.

The current study included 108 DIPP children who had tested positive for f-GADA in the first autoantibody-positive sample during the follow-up. In addition, 31 children positive for f-GADA who had been analyzed for GAD epitope-specific autoantibodies in an earlier DIPP substudy were included (4). Out of the 108 children, 5 had participated in the earlier study. Among the 31 children, 2 had missing data on t-GADA because their latest samples analyzed for epitope-specific GADA had run out and were not available for the analysis. These 2 children were excluded from the current analysis. Accordingly, the final study cohort included 137 DIPP children, of whom 34 had available data on epitope-specific GADA.

#### DIABIMMUNE

DIABIMMUNE is a prospective observational study aimed at testing the hygiene hypothesis in T1D (12). The study was carried out in 3 neighboring countries—Finland, Estonia, and Russia—that differ conspicuously in the incidence of T1D and other immune-mediated diseases.

The DIABIMMUNE Birth Cohort (BC) includes children born between September 2008 and February 2011 in Finland, Estonia, and Russian Karelia, recruited from the general population based on HLA screening from cord blood. The BC participants carried HLA genotypes conferring high, moderate, or slightly increased risk for T1D. They were observed from birth until the age of 3 years and attended study visits including clinical examination and blood sampling at the age of 3, 6, 12, 18, 24, and 36 months. The families of the participants continuously recorded information on the child’s health and lifestyle in the DIABIMMUNE diary, and the data were checked and entered into the database at each follow-up visit. Biological samples were obtained from the participants regularly (12). Cord blood and venous blood samples collected at the follow-up visits were analyzed for diabetes-associated autoantibodies. The current study included 30 children from the BC who had tested positive for f-GADA in at least 1 sample during the follow-up.

Children in the DIABIMMUNE Young Children Cohort (YCC) were recruited from the general population at the age of 36 months to participate in follow-up until the age of 60 months. The clinical study visits took place at the age of 36 and 60 months, unless the child tested positive for islet autoantibodies, tissue transglutaminase antibodies, or at least 2 allergen-specific IgE responses (≥0.35 kU/L) out of 8 tested. Such children and their matched controls were seen also at the age of 48 months. The study set out to monitor the development of T1D, islet autoantibodies, celiac disease, allergies, and infections and to analyze regulatory T-cell function, epigenetic factors, and the gut microbiome. The current study included 65 children from the YCC who had tested positive for f-GADA in at least 1 sample during the follow-up.

#### Early Dietary Intervention and Later Signs of Beta-Cell Autoimmunity

The Early Dietary Intervention and Later Signs of Beta-Cell Autoimmunity (EDIA) study is a clinical trial conducted in the Tampere University Hospital aimed at comparing the effect of weaning to an extensively hydrolyzed casein formula with a conventional cow’s milk-based formula on intestinal permeability, gut microbiota, Th17-mediated immunity, and serum metabolome and proteome (13). The mothers pregnant with a baby to be born in the Tampere University Hospital area were invited to participate in the EDIA study between March 2013 and August 2015. The newborn infants of the participating families were screened for T1D-associated HLA genotypes from cord blood. Families with an HLA-predisposed infant were invited to participate in a 9-month dietary intervention trial. The participants were randomized to be weaned either to an extensively hydrolyzed formula or a conventional formula. During the 9-month intervention period, cow’s milk proteins, beef, and veal were excluded from the infant’s diet. Exclusive breastfeeding was encouraged to be continued as long as possible, and the decision to wean the baby from breast milk and to introduce the study formula was left to the mother.

Altogether 73 children participated in the EDIA follow-up. Venous blood samples were obtained at the age of 3, 6, 9, and 12 months. The infants were monitored to the age of 12 months, after which the families were invited to continue in the DIPP study. All children from EDIA were included in the current study.

### Genetic Screening

#### Analyses of HLA genotypes

Genotyping for the *HLA-DR-DQ* haplotypes was performed by using PCR-based hybridization methods applying lanthanide-labeled probes and time-resolved fluorometry, as previously described (14). In the DIPP study, originally newborn infants carrying the high-risk HLA-DQB1*02/*03:02 or moderate-risk HLA*DQB1*03:02/x (x is not *02, *03:01, or *06:02) genotypes were invited to participate in the study follow-up (10). Later on, more detailed genotyping was performed and full-house DQB1 typing and further DQA1 and DR4 subtyping was performed when relevant for identifying the HLA-associated T1D risk (1). Participants in the EDIA study were screened from cord blood and recruited to the trial if they carried the *DR3-DQ2* (DQA1*05-DQB1*02) and/or *DR4-DQ8* (DRB1*04:01/2/4/5/8-DQB1*03:02/4) genotype in the absence of any protective haplotypes (7). In the DIABIMMUNE BC, the eligible children carried the same HLA genotypes as in EDIA. The participants in the FPDR and DIABIMMUNE YCC were analyzed for HLA genotypes but their HLA genotype was not used as an inclusion criterion. All participants with missing HLA data (n = 31) were excluded from the current study population.

#### Classification of HLA-conferred disease risk

HLA genotypes were classified into six categories according to T1D risk, as previously described (7). The risk categories were further classified into 2 groups: (1) HLA genotypes conferring slightly, moderately, or strongly increased risk for T1D and (2) neutral HLA genotypes and genotypes associated with slightly or strongly decreased risk for T1D (7).

In 1 FPDR participant, the risk category and the presence of the predisposing *DR3-DQ2* haplotype could not be defined. This person carried the *DR4-DQ8* haplotype and the DQB1*02 allele in the other haplotype, but it could not be determined whether the other haplotype was *DR3-DQ2* because data on the *DRB1* and *DQA1* alleles were missing. In any case, the HLA-conferred risk for T1D was increased in this individual (7).

### Autoantibody Analyses

#### Autoantibody samples

For children in the EDIA, YCC, and BC cohorts, the latest autoantibody sample available was selected for the analysis. In the case of the DIPP study, the first autoantibody-positive sample of participants positive for f-GADA at initial seroconversion was analyzed, except for the 31 children previously analyzed for GAD epitope responses in whom the last available sample with data on epitope-specific GADA was used (4). For participants in the FPDR, the autoantibody sample obtained at the diagnosis of the index child was analyzed.

#### Autoantibodies to full-length GAD(1**-**585) and N-terminally truncated GAD(96-585)

All samples were analyzed for f-GADA and t-GADA by using specific radiobinding assays, as previously described (3,15). The autoantibody titers were expressed in relative units (RU) calculated by applying the MultiCalc Software based on a standard curve run on each assay plate (Perkin Elmer Life Sciences Wallac, Turku, Finland). The cutoff limit for autoantibody positivity in the GADA assays (5.36 RU) was set at the 99th percentile of 373 nondiabetic Finnish children and adolescents. The sensitivity of the f-GADA assay was 64% to 88%, and the specificity was 94% to 99%, respectively, based on the Diabetes Autoantibody Standardization Program and the Islet Autoantibody Standardization Program results in 2010-2018. The corresponding performance characteristics for the t-GADA assay were 70% to 78% and 94% to 100% based on the 2012-2020 standardization rounds.

#### GAD65 epitope-specific autoantibodies

Epitope-specific GADA were analyzed by applying 3 GAD65/GAD67 chimeras, as previously described (3). The cutoff limits for positivity in the epitope assays were set at the 99th percentile values among 104 nondiabetic Finnish children and adolescents: 0.86 RU for the N-epitope-specific antibodies, 1.51 RU for the M-epitope-specific antibodies, and 1.59 RU for the C-terminal epitope-specific antibodies (3).

In the current study, epitope-specific GADA were analyzed from the samples of the 90 FPDR index children and the 65 children in the YCC. In addition, data from 34 DIPP participants previously analyzed for epitope-specific GADA were included in the analysis (4). For the DIPP participants, the last available sample with data on epitope-specific GADA was used for the analyses of GAD epitope responses.

#### Other biochemical autoantibodies

IAA, IA-2A, and ZnT8A were analyzed using radiobinding assays, as described earlier (16-18). The analyses of biochemical autoantibodies were carried out in the PEDIA Laboratory, University of Helsinki, Helsinki, Finland, except for the analyses of IAA, GADA, and IA-2A in the DIPP study, which were performed in the Research Laboratory, Department of Pediatrics, University of Oulu, Oulu, Finland. The cutoff limits for autoantibody positivity were as follows: 1.57 RU for IAA, 0.77 RU for IA-2A, 0.50 RU for ZnT8A (ZnT8 plasmid clone 6.2), and 0.61 RU for ZnT8A (ZnT8 plasmid clone 4.1) in the PEDIA laboratory and 3.48 RU for IAA, and 0.43 RU for IA-2A in the Oulu laboratory.

#### Islet cell antibodies

ICA were analyzed by using a standardized immunofluorescence-based staining method, as previously described (19). The detection limit for ICA positivity was 2.5 Juvenile Diabetes Foundation units. ICA were analyzed in the Research Laboratory, Department of Pediatrics, University of Oulu, Oulu, Finland.

### Definitions

T1D was diagnosed according to the World Health Organization criteria (20).

### Statistical Analyses

IBM SPSS software for Macintosh (version 25.0, Armonk, New York, NY, USA) was applied to perform statistical analyses. CI was set at 95%, and the statistical significance at *P* < 0.05 (2-sided). Statistical differences were tested by using Pearson’s χ ^2^ test, Fisher’s exact test, and the Mann-Whitney U test, when applicable. Statistical correlations between nonparametric variables were tested by using Spearman’s rank correlation coefficient, except for the f- and t-GADA titers, which were analyzed by using linear regression. The sensitivities, specificities, positive predictive value (PPV), and negative predictive value (NPV) were determined, as previously described (21). The corresponding CIs were assessed by using a web-based calculator (http://statpages.info/ctab2x2.html).

### Ethical Approval

The local ethics committees of the participating study centers have approved the DIPP, FPDR, DIABIMMUNE, and EDIA study protocols. All study procedures were carried out according to the principles of the Declaration of Helsinki. The legal caretakers of underaged participants gave their written informed consent for the HLA screening and for participation in the study procedures.

## Results

### Autoantibodies to N-terminally Truncated GAD as a Marker for T1D

T1D had been diagnosed in 174 (22.9%) participants (Table 1). Among individuals positive for f-GADA, the frequency of T1D was significantly higher among those who tested positive for t-GADA (n = 451) than among those who tested negative (29% vs 2%; *P* < 0.001). Screening for t-GADA improved the specificity (46%) and PPV (30%) of predicting T1D relative to screening for f-GADA (15% and 21%, respectively) (Table 2). The sensitivity for both assays was similar (79% and 76%, respectively). The combination of t-GADA and f-GADA did not improve the predictive characteristics further compared to t-GADA alone. No sex difference emerged in the frequency of positivity for f-GADA or t-GADA (data not shown).

**Table 1.** Clinical characteristics in the study population

		Cohort					
Clinical characteristic	Total	FPDR, index children	FPDR, family members	EDIA	DIPP	DIABIMMUNE, YCC	DIABIMMUNE, BC
Inclusion criteria in the current study population		f-GADA and/or ICA positive at T1D diagnosis	f-GADA and/or ICA positive at diagnosis of index child	All EDIA children included	(1) f-GADA positive in the first AAB-positive sample during DIPP follow-up and/or (2) the first 36 DIPP children positive for f-GADA in ≥2 samples preclinically, data available on t-GADA and epitope-specific GADA (n = 34)	f-GADA positive ≥1 sample during DIABIMMUNE follow-up	f-GADA positive in ≥1 sample during DIABIMMUNE follow-up
Participants, n	760	90	365	73	137	65	30
Sex, male	408 (53.7)	54 (60.0)	183 (50.1)	35 (47.9)	79 (57.7)	34 (52.3)	23 (76.7)
HLA risk group							
Increased risk	521 (72.4)	70 (77.8)	219 (60.0)	73 (100.0)	131 (95.6)	27 (41.5)	30 (100.0)
Neutral or protective genotype	210 (27.6)	20 (22.2)	146 (40.0)	0 (0)	6 (4.4)	38 (58.5)	0 (0)
HLA haplotypes							
*DR3-DQ2*^a^	276 (36.3)	26 (28.9)	117 (32.1)	34 (46.6)	56 (40.9)	26 (40.0)	17 (56.7)
*DR4-DQ8*^b^	483 (63.6)	68 (75.6)	197 (54.0)	59 (80.8)	126 (92.0)	15 (23.1)	18 (60.0)
Type 1 diabetes	174 (22.9)	90 (100.0)	2 (0.5)	1 (1.4)	64 (46.7)	8 (12.3)	9 (30.0)
AAB positivity							
Full-length GADA	631 (83.0)	55 (61.1)	364 (99.7)	2 (2.7)	134 (97.8)	46 (70.8)	30 (100.0)
Truncated GADA	451 (59.3)	58 (64.4)	220 (60.3)	1 (1.4)	118 (86.1)	36 (55.4)	18 (60.0)
Multiple (≥2) AABs	260 (34.2)	84 (93.3)	75 (20.5)	2 (2.7)	68 (49.6)	17 (26.2)	14 (46.7)
Multiple (≥2) biochemical AABs	143 (18.8)	71 (78.9)	6 (1.6)	2 (2.7)	37 (27.0)	13 (20.0)	14 (46.7)
ICA	246 (43.3)	82 (91.1)	71 (19.5)	2 (2.7)	65 (47.4)	13 (20.0)	13 (43.3)
IAA	103 (13.6)	38 (42.2)	6 (1.6)	5 (6.8)	31 (22.6)	10 (15.4)	13 (43.3)
IA-2A	96 (12.6)	65 (72.2)	0 (0)	1 (1.4)	17 (12.4)	7 (10.8)	6 (20.0)
ZnT8A^c^	98 (14.1)	58 (64.4)	0 (0)	1 (1.4)	20 (15.2)	10 (15.4)	9 (30.0)
f-GADA and t-GADA	439 (57.8)	53 (58.9)	220 (60.3)	1 (1.4)	116 (84.7)	31 (47.7)	18 (60.0)
f-GADA and ICA	206 (27.1)	49 (54.4)	71 (19.5)	1 (1.4)	62 (45.3)	10 (15.4)	13 (43.3)
f-GADA, ICA, and t-GADA	186 (24.5)	47 (52.2)	61 (16.7)	1 (1.4)	55 (40.1)	10 (15.4)	12 (40.0)
Age at sampling, years	9.0 (0.2-61.5)	9.7 (1.5-16.2)	35.0 (0.5-61.5)	1.0 (0.3-1.1)	4.0 (0.5-14.2)	5.1 (2.8-6.8)	2.1 (0.2-3.8)
Age at diagnosis, years	8.5 (0.8-19.7)	9.6 (1.3-16.2)	15.3 (12.2-18.4)	1.9 (-)	7.7 (0.8-19.7)	5.5 (2.6-8.9)	6.1 (2.4-8.7)
f-GADA titer, RU	11.6 (0.0-3524.8)	8.2 (0.0-204.6)	15.0 (0.0-304.9)	0.0 (0.0-24.9)	21.4 (1.3-228.1)	8.2 (0.0-3524.8)	10.2 (5.5-1056.6)
t-GADA titer, RU	9.0 (0.0-3562.5)	9.2 (0.0-209.6)	9.4 (0.0-225.9)	0.0 (0.0-52.2)	27.3 (0.2-224.4)	7.9 (0.0-3562.5)	10.0 (0.0-558.5)
ICA titer, JDFU	0.0 (0.0-2048.0)	23.0 (0.0-374.0)	0.0 (0.0-2048.0)	0.0 (0.0-513.0)	0.0 (0.0-436.0)	0.0 (0.0-2048.0)	0.0 (0.0-2048.0)
AAB titers^d^							
f-GADA titer, RU	16.2 (5.4-3524.8)	19.2 (5.5-204.6)	15.1 (5.4-304.9)	17.2 (9.5-24.9)	26.4 (5.5-228.1)	11.2 (6.2-3524.8)	10.2 (5.5-1056.6)
t-GADA titer, RU	26.9 (5.5-3562.5)	17.4 (6.1-209.6)	28.9 (5.5-225.9)	52.2 (-)	40.7 (5.6-224.4)	18.8 (5.7-3562.5)	17.9 (7.1-558.5)
ICA titer, JDFU	16.0 (2.5-2048.0)	44.0 (3.0-374.0)	9.0 (3.0-2048.0)	289.0 (65.0-513.0)	8.0 (2.5-436.0)	64.0 (6.0-2048.0)	256.0 (4.0-2048.0)

*Data are given as n (%) or median (range), unless otherwise noted.

^a^ DRB1*03:01-DQA1*05(:01)-DQB1*02. Data available for 759 participants.

^b^ DRB1*04:01/02/04/05/08-DQA1*03(:01)-DQB1*03:02(/04). Data available for 760 participants.

^c^ZnT8A data available for 90 FPDR index children, 303 FPDR relatives, 132 DIPP children, and all EDIA, YCC, and BC participants.

^d^Among individuals positive for the specific autoantibody reactivity.

Abbreviations: AAB, autoantibody; BC, birth cohort; DIPP, Type 1 Diabetes Prediction and Prevention Study; EDIA , Early Dietary Intervention and Later Signs of Beta-cell Autoimmunity; f-GADA, autoantibodies to full-length GAD(1-585); FPDR, Finnish Pediatric Diabetes Register; IA-2A, autoantibodies to islet antigen-2; IAA, insulin autoantibodies; ICA, islet cell antibodies; JDFU, Juvenile Diabetes Foundation Unit; RU, relative unit; T1D, type 1 diabetes; t-GADA, autoantibodies to N-terminally truncated GAD(96-585); YCC, Young Children Cohort; ZnT8A, autoantibodies to zinc transporter 8.

**Table 2.** Positivity for t-GADA, f-GADA, and combined positivity for t-GADA and f-GADA among progressors and nonprogressors in the study population (n = 760), and the sensitivities, specificities, and positive and negative predictive values of positivity for t-GADA, f-GADA, and combined positivity for t-GADA and f-GADA for type 1 diabetes

Autoantibody positivity	Progressors	Nonprogressors	*P*-value	Sensitivity	Specificity	PPV	NPV
	n (%)	% (95% CI)					
t-GADA	137 (79)	314 (54)	<0.001	79 (73-84)	46 (45-48)	30 (28-32)	88 (85-91)
f-GADA	132 (76)	499 (85)	0.004	76 (70-81)	15 (13-16)	21 (19-22)	67 (60-75)
f-GADA and t-GADA	129 (74)	310 (53)	<0.001	74 (68-80)	47 (45-49)	29 (27-32)	86 (83-89)

Abbreviations: f-GADA, autoantibodies to full-length GAD(1-585); NPV, negative predictive value; PPV, positive predictive value; t-GADA, autoantibodies to N-terminally truncated GAD(96-585).

### HLA Associations of Autoantibodies to N-terminally Truncated GAD

Among the 631 participants positive for f-GADA, those who tested positive for t-GADA more frequently carried HLA genotypes conferring increased risk for T1D than those who tested negative for t-GADA (77% vs 53%; *P* < 0.001). Also the high-risk *DR3-DQ2/DR4-DQ8* genotype was present more frequently among those who tested positive for t-GADA (19% vs 9%; *P* = 0.002). These observations held true as well when the analysis was restricted to participants under the age of 20 years (81% vs 57%; *P* < 0.001 and 23% vs 12%; *P* = 0.03, respectively). In the whole study population, positivity for t-GADA was more common among participants with predisposing HLA genotypes than in those with neutral or protective genotypes (63% vs 50%; *P* = 0.001), but this difference did not remain significant in participants younger than 20 years (61% vs 53%; *P* = 0.13).

Positivity for t-GADA was associated with the predisposing *DR4-DQ8* haplotype when compared with negativity for this haplotype (64% vs 51%; *P* < 0.001). However, no difference was observed in the frequency of positivity for t-GADA between individuals with and without the predisposing *DR3-DQ2* haplotype (60% vs 59%; *P* = 0.76). Subjects who had GADA as the only biochemical autoantibody and tested positive for t-GADA more frequently carried the *DR4-DQ8* haplotype than those who tested negative for t-GADA (65% vs 46%; *P* < 0.001), while no difference was seen in the prevalence of the *DR3-DQ2* haplotype (37% vs 34%; *P* = 0.45).

In subjects with the *DR4-DQ8/x* genotype and either DRB1*04:01 or DRB1*04:04 allele in the *DR4-DQ8* haplotype, positivity for t-GADA was more common among those positive for the DRB1*04:04 allele than among those negative for this allele (71% vs 61%; *P* = 0.048). In contrast, positivity for t-GADA was less frequent among those carrying the DRB1*04:01 allele than among those without this allele (61% vs 73%; *P* = 0.02).

### HLA Associations of GAD Epitope-specific Autoantibodies

Among the 189 children analyzed for epitope-specific GADA, those carrying predisposing HLA genotypes had more often autoantibodies against the M-epitope than those with neutral or protective genotypes (Table 3). However, no significant difference emerged in the frequency of positivity for autoantibodies against the N- or C-terminal epitopes between the HLA groups. Isolated positivity for autoantibodies against the N-epitope was slightly more common among carriers of neutral or protective HLA genotypes than among those with predisposing genotypes (Table 3).

**Table 3.** Positivity for GAD epitope-specific autoantibodies in the HLA groups among the 189 children with available data on epitope-specific GADA

Whole study population (n=189)	Autoantibody positivity	Predisposing HLA genotypes, n (%) (n = 130)	Neutral or protective HLA genotypes, n (%) (n = 59)	*P*-value
	N-epitope (n = 24)	13 (10.0)	11 (18.6)	0.10
	M-epitope (n = 70)	58 (44.6)	12 (20.3)	0.001
	C-epitope (n = 53)	41 (31.5)	12 (20.3)	0.11
	Isolated N-epitope (n = 13)	5 (3.8)	8 (13.6)	0.01
	Isolated M-epitope (n = 21)	16 (12.3)	5 (8.5)	0.44
	Isolated C-epitope (n = 4)	0 (0)	4 (6.8)	0.009
Participants positive for f-GADA (n = 132)				
	N-epitope (n = 21)	12 (13.5)	9 (20.9)	0.27
	M-epitope (n = 70)	58 (65.2)	12 (27.9)	<0.001
	C-epitope (n = 51)	41 (46.1)	10 (23.3)	0.01
	Isolated N-epitope (n = 11)	4 (4.5)	7 (16.3)	0.04
	Isolated M-epitope (n = 21)	16 (18.0)	5 (11.6)	0.35
	Isolated C-epitope (n = 3)	0 (0)	3 (7.0)	0.03

Abbreviations: f-GADA, autoantibodies to full-length GAD(1-585); GAD, glutamic acid decarboxylase; GADA, autoantibodies to glutamic acid decarboxylase; NPV, negative predictive value; PPV, positive predictive values; t-GADA, autoantibodies to N-terminally truncated GAD(96-585); HLA, human leukocyte antigen.

Among individuals positive for f-GADA, predisposing HLA genotypes were found more often among those with autoantibodies against the M-epitope (83% vs 50%; *P* < 0.001) and/or C-epitope (80% vs 59%; *P* = 0.01) than among those negative for autoantibodies against these epitopes, respectively. Autoantibodies against both the M-epitope (47% vs 22%; *P* < 0.001) and/or the C-epitope (35% vs 16%; *P* = 0.005) were more common among subjects positive for the *DR4-DQ8* haplotype than among those negative for this haplotype. The former were also more frequent among individuals positive for the *DR3-DQ2*. No significant difference emerged in the frequency of the C-epitope-specific autoantibodies between these groups (35 vs 24%; *P* = 0.10).

In subjects with the *DR4-DQ8*/x genotype and either DRB1*04:01 or DRB1*04:04 allele in the *DR4-DQ8* haplotype, autoantibodies against the M-epitope were more common among those carrying the DRB1*04:04 allele than among those negative for this allele (63% vs 41%; *P* = 0.03) but less frequent among those positive for the DRB1*04:01 allele than among those negative for the DRB1*04:01 allele (42% vs 64%; *P* = 0.04). The same applied to autoantibodies against the C-epitope that were associated with the presence of the DRB1*04:04 allele (54% vs 28%; *P* = 0.007) but inversely associated with the presence of the DRB1*04:01 allele (30% vs 54%; *P* = 0.03).

No difference emerged in the prevalence of the predisposing HLA genotypes in relation to positivity for N-epitope-specific GADA (57% vs 69%; *P* = 0.27). However, subjects with isolated N-epitope positivity more often carried neutral or protective HLA genotypes than the rest of the individuals positive for f-GADA (64% vs 30%; *P* = 0.04).

### Positivity for Multiple Autoantibodies

Positivity for multiple (≥2) biochemical autoantibodies emerged more commonly among participants positive for both f-GADA and t-GADA than among those with only f-GADA [26.2% (n = 115) vs 1.6% (n = 3); *P* < 0.001]. When ICA was introduced into the analysis, positivity for 2 or more autoantibodies was nearly 5 times more common among those who tested positive for t-GADA relative to those positive for f-GADA alone [46.4% (n = 202) vs 10.4% (n = 20); *P* < 0.001].

### ICA Reactivity

Among participants positive for f-GADA, those who tested positive for ICA presented more often with positivity for t-GADA (90% vs 60%; *P* < 0.001) and had higher titers of t-GADA than ICA-negative individuals (median 48.2 vs 8.4 RU; *P* < 0.001). Among ICA-positive subjects, positivity for f-GADA was mostly derived from reactivity toward the N-terminally truncated GAD (90%), whereas only 20 (10%) ICA-positive subjects tested positive for f-GADA in the absence of t-GADA (*P* < 0.001). Furthermore, participants simultaneously positive for ICA and f-GADA had higher titers of ICA in the presence of t-GADA than if they remained negative for t-GADA [16.0 (n = 186) vs 4.5 (n = 20) Juvenile Diabetes Foundation units; *P* < 0.001]. In the whole study population, there was a positive correlation between the titers of ICA and t-GADA (Spearman correlation coefficient 0.415; *P* < 0.001) and between the titers of ICA and f-GADA (Spearman correlation coefficient 0.266; *P* < 0.001).

### Titers of GAD Autoantibodies

**Figure 1.**
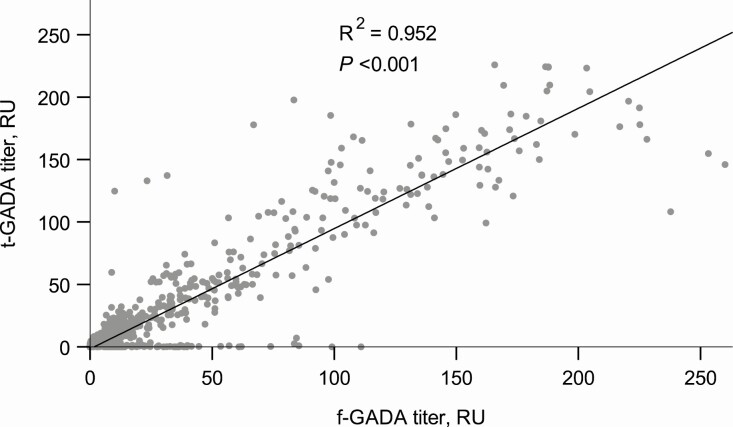
Association between f-GADA and t-GADA titers.

Since a broader range of GAD epitope-specific responses has been observed along with higher f-GADA titers, we analyzed the association between t-GADA positivity and f-GADA titer (22). The titers of f-GADA were significantly higher among subjects who tested positive for t-GADA than among those who tested negative (median 26.4 vs 7.3 RU; *P* < 0.001). The titers of f-GADA and t-GADA were strongly correlated (r = 0.976, *P* < 0.001) (Fig. 1).

### Age at Diagnosis

No significant correlation emerged between t-GADA titer and age at diagnosis of T1D (Spearman correlation coefficient −0.030; *P* = 0.69), nor was positivity for t-GADA related to the age at diagnosis (median 8.6 vs 8.5 years; *P* = 0.61).

## Discussion

The increasing incidence of T1D has emphasized the need to develop improved tools for disease prediction and to identify the underlying mechanisms causing T1D (23). In this cross-sectional study combining data from 6 study cohorts, we examined the value of autoantibodies to N-terminally truncated GAD(96-585) as a biomarker for T1D. We demonstrated an association between autoantibody responses to epitopes within the N-terminally truncated GAD and HLA class II genotypes, conferring increased risk for T1D, and between isolated positivity for the N-terminal epitope and neutral or protective HLA genotypes. Adding to earlier reports on HLA associations of truncated GAD autoantibodies (8), the current study reports at least 2 new findings: (1) the higher frequency of t-GADA in individuals carrying the *DR4-DQ8*/x genotype and specifically the DRB1*04:04 allele in the *DR4-DQ8* haplotype compared with those carrying the DRB1*04:01 allele in this haplotype and (2) the higher frequency of M-epitope–specific GADA among individuals positive for the haplotypes confering the highest HLA risk for T1D (*DR4-DQ8* and *DR3-DQ2*) relative to those without such haplotypes.

As expected, positivity for t-GADA was associated with clinical T1D. This was evident from the improved specificity and PPV for T1D when t-GADA positivity was compared with f-GADA positivity. The sensitivity for both assays remained quite similar, which is in accordance with earlier reports (2). Compared to f-GADA, t-GADA are less frequently seen among healthy subjects, possibly due to reduced presentation of conformational epitopes by the N-terminally truncated GAD (24).

Epitope spreading is a frequent phenomenon during the prediabetic disease process (25). The initial autoantibody response against GAD has been shown to occur mostly against the M- and C-terminal epitopes among progressors to T1D whereas isolated responses toward the N-epitope do not increase the risk for T1D (3,4,26,27). The GAD-specific autoimmune response usually spreads from the M-region to the C-terminal, rather than the N-terminal (22,25). What drives this epitope spreading has not, however, been fully understood. In the current study, we observed distinct distributions of HLA class II genotypes among subjects positive for GAD epitope-specific autoantibodies, which might explain some of the epitope spreading related to the risk of T1D. Positivity for t-GADA was associated with HLA genotypes predisposing to T1D, especially the *HLA-DR4-DQ8*/x genotype, while positivity for f-GADA without t-GADA was associated with a higher prevalence of neutral or protective HLA genotypes. Notably, especially the predisposing *HLA-DR4-DQ8* haplotype and more specifically the DRB1*04:04 allele in this haplotype were associated with autoantibodies against epitopes within GAD(96-585). In the Finnish population, the DRB1*04:04 allele was recently associated with GADA as the preferential first islet autoantibody while the other common DR4 allele, DRB1*04:01, was preferentially associated with IAA-initiated autoimmunity (28). These findings might suggest that HLA class II-mediated antigen presentation of peptides derived from GAD(96-585) or structurally highly similar peptides represents an important insult in the pathogenesis of T1D.

Among subjects positive for f-GADA, autoantibodies to the C-epitope and especially the M-epitope emerged significantly more frequently among individuals carrying HLA genotypes predisposing to T1D than among those carrying neutral or protective genotypes. Again, isolated autoantibodies to the N-epitope were associated with neutral or protective HLA genotypes. This supports the previous observations that the primary GAD epitope associated with T1D might be located within the middle region of the protein (4,26). In adult-onset diabetes, t-GADA increase the risk for insulin therapy, indicating that autoantibodies to the M- and C-epitopes might reflect more aggressive beta-cell destruction (29). Recently, it was also reported that the first 142 amino acids of GAD do not contribute to the generation of autoantibodies associated with T1D (30).

Considering the critical role of environmental factors in T1D, the current observations linking the predisposing HLA genotypes with autoimmunity against the GAD amino acids 96-585 suggests that a specific sequence within this part of the protein might cross-react with a foreign immunogenic peptide. In fact, a high degree of homology has been observed between GAD(247-280) and the Coxsackievirus protein P2-C (31) and between GAD(115-127) and the immunogenic rotavirus protein VP7 (32). Both Coxsackie- and rotaviruses have been associated with islet autoimmunity and T1D, although the observed associations might not reflect a causal relationship (33-36). Furthermore, the truncated GAD(96-585) might comprise a potential target for autoantigen-specific immunomodulatory therapies aimed at inducing tolerance. Previously, the administration of full-length GAD-Alum to children with multiple islet autoantibodies proved safe but did not affect progression to T1D (37). Also, tertiary prevention trials in the United States and Sweden failed to demonstrate any efficacy of full-length GAD-Alum, but in the Swedish study the treatment showed a favorable effect on the preservation of residual beta-cell function (38-40).

Finally, we explored the association between t-GADA and other islet autoantibodies. As expected, positivity for multiple biochemical autoantibodies emerged nearly exclusively in participants positive for t-GADA when compared to those positive for f-GADA alone. Similarly, most of the positivity for f-GADA among ICA-positive subjects was derived from positivity for t-GADA. In addition, high ICA titers were associated with positivity for t-GADA. This might partly explain the previous observations that high ICA titers are mostly seen together with other islet autoantibodies and strongly increase the risk of clinical T1D (5,41). ICA reactivity is in part derived from autoantibody responses toward GAD, other islet autoantigens, and yet unidentified antigens (42,43). In a recent 15-year DIPP update, inverse seroconversions and fluctuations of ICA positivity rarely occurred among progressors to T1D but often emerged among nonprogressors (41). In that study, there were no ICA-negative individuals among children with multiple autoantibodies, which is interesting, since positivity for multiple autoantibodies strongly indicates progressive islet autoimmunity (44). It is enticing to consider whether reactivity to epitopes within GAD(96-585) might contribute to the stability, high titers, and disease association of ICA among progressors to T1D. Moreover, GAD epitope-specific responses might explain some of the diversity in the pace of disease progression (11,45). Autoantibodies to the M- and C-terminal epitopes have been previously associated with positivity for multiple autoantibodies, while reactivity to the N-epitope indicates isolated GADA positivity (27). The current findings support the idea that t-GADA might be a suitable marker for progressive islet autoimmunity.

A major limitation in the current study is that no data were available on the development of T1D for the FPDR family members older than 16 years, which probably affected the sensitivity and specificity of t-GADA and f-GADA assays for T1D. Furthermore, the predisposing HLA genotypes were probably enriched in participants selected for the analysis of t-GADA based on positivity for f-GADA and/or ICA. This may have affected the observed distributions of HLA genotypes. Some individuals positive for t-GADA without f-GADA and/or ICA may have been missed, although the proportion of such individuals seems to be very limited. Accordingly, the association between the predisposing HLA genotypes and t-GADA positivity needs to be confirmed in future studies. Moreover, 50% of the participants carrying neutral or protective HLA genotypes were adult FPDR relatives. This might limit the generalizability of the current observations to the pediatric population. In the GAD epitope-specific analyses, the number of children with available data was modest but enabled statistical testing.

To conclude, the use of t-GADA improves the specificity of identifying T1D without loss of sensitivity relative to autoantibodies to full-length GAD in a heterogeneous population comprising newly diagnosed children with T1D, children with HLA predisposition to T1D, FDRs of children with T1D, and GADA-positive children from the general population. Autoantibody responses to the N-terminally truncated GAD, especially toward the M-epitope, are associated with HLA class II genotypes predisposing to T1D, while isolated positivity for N-epitope-specific GADA is associated with neutral or protective HLA genotypes. These observations propose that the use of t-GADA instead of f-GADA would significantly improve the screening for risk of T1D and the selection of participants for follow-up studies, especially if combined with screening for T1D risk-associated *HLA-DR-DQ* haplotypes. However, the observed HLA-associations remain to be confirmed in future studies.

## Data Availability

The data generated and analyzed during the current study are available from the corresponding author on reasonable request.

## Notes

***Author Contributions*** P.M.P. collected and analyzed the data and contributed to the study conception and design, data acquisition, analysis, and interpretation and wrote the first version of the manuscript. T.H. was in charge of the autoantibody laboratory analyzing N-terminally truncated GAD_65_ autoantibodies and contributed to discussion. H.S. contributed to study design and discussion and edited the manuscript. M.K., J.I., J.T., and R.V. generated data, contributed to discussions, and revised the manuscript for important intellectual content. M.K. designed the study, is the guarantor of this work, had full access to all the data in this work, and takes responsibility for data integrity and accuracy of the data analysis.

***Funding*** This work was supported by the following grants: Juvenile Diabetes Research Foundation (grants 1-SRA-2016-342-M-R, 1-SRA-2019-732-M-B); European Union (grant BMH4-CT98-3314 and FP7/2007-2013 grant 202063); Novo Nordisk Foundation; Academy of Finland (Decision No 292538) and Centre of Excellence in Molecular Systems Immunology and Physiology Research 2012-2017 (Decision No. 250114); Special Research Funds for University Hospitals in Finland; Diabetes Research Foundation, Finland; Sigrid Jusélius Foundation, Finland; Finska Läkaresällskapet, Finland; Biomedicum Helsinki Foundation, Finland; Orion Research Foundation sr, Finland; Pediatric Research Foundation, Finland; and Yrjö Jahnsson Foundation, Finland.

***Disclosure Summary*** The authors have nothing to disclose.
